# Managing Mental Health Disorders Resulting from Trauma through Yoga: A Review

**DOI:** 10.1155/2012/401513

**Published:** 2012-06-19

**Authors:** Shirley Telles, Nilkamal Singh, Acharya Balkrishna

**Affiliations:** Department of Yoga Research, Patanjali Research Foundation, Haridwar, Uttarakhand 249408, India

## Abstract

There are many and varied types of trauma. The extent to which trauma influences the mental health of an individual depends on the nature of trauma, as well as on the individual's coping capabilities. Often trauma is followed by depression, anxiety, and PTSD. As the pharmacological remedies for these conditions often have undesirable side-effects, nonpharmacological remedies are thought of as a possible add-on treatment. Yoga is one such mind-body intervention. This paper covers eleven studies indexed in PubMed, in which mental health disorders resulting from trauma were managed through yoga including meditation. The aim was to evaluate the use of yoga in managing trauma-related depression, anxiety, PTSD and physiological stress following exposure to natural calamities, war, interpersonal violence, and incarceration in a correctional facility. An attempt has also been made to explore possible mechanisms underlying benefits seen. As most of these studies were not done on persons exposed to trauma that had practiced yoga, this is a definite area for further research.

## 1. Introduction

The definitions of trauma are many and varied. One description states that an event is traumatic if it is extremely upsetting and at least temporarily overwhelms the individual's inner resources [1]. In the United States, surveys of the general population suggest that at least half of all the adults have experienced at least one major traumatic stressor [2, 3].

## 2. Trauma: Types of Trauma and Their Physiological Consequences

The types of trauma include (i) natural disasters, (ii) mass interpersonal violence, (iii) large-scale transportation accidents, (iv) house or other domestic fires, (v) motor vehicle accidents, (vi) rape and sexual assault, (vii) stranger physical assault, (viii) partner battery and emotional abuse, (ix) torture, (x) war, (xi) child abuse, (xii) exposure of emergency workers to trauma, and (xiii) major accident or illness. In listing traumas separately there may be an erroneous impression that such traumas are independent of one another. This is applicable to noninterpersonal traumas such as natural disasters or house fires. However, it is also recognized that victims of interpersonal traumas are at greater risk of additional interpersonal traumas. For example those who have experienced child abuse are more likely to be victimized as adults [4, 5].

People respond to trauma in different ways. When exposed to the same trauma, some people develop posttraumatic stress disorder, whereas others are less affected or respond with symptoms such as depression or generalized anxiety [6].

It is well recognized that severe psychological trauma causes impairment of the neuroendocrine systems in the body, with sympathetic activation and suppression of the parasympathetic nervous system. There is also an increase in the level of circulating cortisol which has adverse effects on different systems. Severe trauma in early childhood especially has serious consequences. It can affect all aspects of development, including cognitive, social, emotional, physical, psychological, and moral development [7]. This has serious consequences in adolescence and can influence adult life as well.

Van der Kolk et al. (1996) described the long-term effects of trauma, which were: generalized hyper-arousal and difficulty in modulating arousal, alterations in neurobiological processes involved in stimulus discrimination, conditioned fear responses to trauma related stimuli, loss of trust and hope, social avoidance, and lack of interest and participation in preparing for the future [8]. With these changes, it is understandable that if the trauma is severe, and if support is inadequate and hereditary factors are also present, a person may develop symptoms of depression.

The pharmacological management of psychological disorders resulting from trauma is best supplemented with nonpharmacological healing techniques which would allow the person to regulate their internal states and response to external stress [9].

The Indian science of living, yoga, includes several practices such as physical postures (*asanas*), voluntary regulated breathing (*pranayamas*), meditation, conscious sensory withdrawal (*pratyahara*), and philosophical principles [10]. 

## 3. Yoga and the Management of the Consequences of Trauma

It is often difficult for people who have been subjected to acute or prolonged trauma to regain a sense of normalcy and balance in their lives [11]. In many cases, psychiatric help is sought [12]. Also, since yoga requires involvement and ultimately trains the person to practice on their own, learning yoga can help a person regain their sense of being in control of their lives, as well as increase their self-dependence. 

Yoga is a nonpharmalogical remedy which has been used to help in managing trauma related to (i) natural disasters, (ii) combat and terrorism, (iii) interpersonal violence, and (iv) being incarcerated in a correctional facility. Studies published in journals indexed in PubMed were reviewed. Those indexed in other bibliographic databases or which did not use yoga, including meditation as an intervention, were excluded.

### 3.1. Yoga and Coping with Natural Disasters

In December 2004, a tsunami occurred in South East Asia [13]. Two studies were conducted to assess the effects of yoga on the survivors. A single group study on 47 tsunami survivors with ages between 28 and 50 years was evaluated to see the effects of a 7-day yoga program [14]. The yoga program included loosening exercises, yoga postures, breathing practices, and guided relaxation for 60 minutes daily. Participants were assessed for self-rated symptoms of stress (viz., fear, anxiety, disturbed sleep, and sadness) using linear analog scales. The electrocardiogram (to assess heart rate variability), breath rate, and skin resistance were recorded using a portable four-channel polygraph. Data were analyzed using paired *t*-tests and all self-rated symptoms significantly reduced along with the breath rate. Effect sizes were not calculated. The three main limitations of the study were (i) the absence of a control group, (ii) use of scales whose reliability and validity had not been established, and (iii) the short duration of the intervention with no long-term follow up. To a large extent, these shortcomings were overcome in the second study which evaluated 183 tsunami survivors who were above the age of 18 years and who scored 50 or above on the Posttraumatic Check List-17 (PCL-17) [15]. They were allocated to three groups. The groups were (i) yoga breath intervention, (ii) yoga breath intervention followed by 3 to 8 hours trauma reduction exposure technique, and (iii) a wait list control group. The participants were assessed at the beginning and end of 24 weeks, as well as intermittently usingthe Posttraumatic Check List (PCL-17) for PTSD and Beck Depression Inventory (BDI-21) for depression. The data were analyzed using a three-way ANOVA. The scores of PCL-17 significantly reduced with yoga breath intervention and yoga breath intervention followed by 3–8 hours trauma reduction exposure technique. This study used scales whose validity and reliability had been established and the participants were followed up for 24 weeks. The effect sizes were not calculated. The only weakness of the study was that the allocation of the participants to three different groups was not random.

A meditation technique called Inner Resources was used in a single study on 20 health workers (aged between 31 and 67 years) who were involved in relief work, 10 weeks after Hurricane Katrina [16]. Meditation was taught as a 4 hour workshop followed by an 8-week home study program. The participants were assessed using disaster exposure questions, PTSD Checklist-Specific Version (PCL-S), the Center for Epidemiological Studies-Depression Scale (CES-D), the 20-item state portion of the State-Trait Anxiety Inventory (STAI-S) and a follow up questionnaire. Intention-to-treat regression analyses and one-sample *t*-tests showed that the persons' PTSD scores assessed by PCL-S and anxiety symptoms assessed by STAI-S significantly decreased with the intervention. Treatment effect size based on Cohen's *d* was calculated for PCL-S total (*d* = .38), CES-D (*d* = .12) and STAI-S (*d* = .45). The limitations of the study were the small sample size and absence of a control group. 

Both the tsunami and the hurricane were unexpected natural disasters. In certain cases, natural disasters occur repeatedly. An example is the floods in the north eastern Indian state of Bihar caused by seasonal rain and a breach in a river [17]. In a cross-sectional study 1289 people (aged between 15 to 85 years) who had been directly exposed to the floods were screened for PTSD and depression using the Screening questionnaire for disaster mental health (SQD) [17]. In this study, no intervention was given. Two factor ANOVAs showed that people over 60 years of age had higher scores of PTSD and depression. From the larger sample of 1289 persons, a small sample of 22 persons (with an age range of 20 to 40 years) were selected based on their willingness to participate in the trial and randomized as two groups, a yoga group and a control group who continued with their regular activities [18]. The yoga intervention was for 7 days and included loosening exercises, yoga postures, breathing exercises, and guided relaxation for 60 minutes everyday. The self-rated symptoms of distress, namely, fear, anxiety, disturbed sleep, and sadness were assessed with linear analog scales, the heart rate variability based on the electrocardiogram and breath rate were recorded with a four channel polygraph. The yoga group showed a significant decrease in sadness while anxiety significantly increased in the control group (paired *t*-tests were used for analyses). Effect sizes were not calculated in this study. Limiting factors were the use of analog scales which have not been validated and the small sample size.

### 3.2. Yoga and Coping with Exposure to Combat and Terrorism

In Kosovo, 139 high school students with ages ranged from 12 to 19 years took part in a single group study in which the 6 week program included meditation, biofeedback, drawings, autogenic training, guided imagery, genograms, movement, and breathing techniques [19]. They were trained in the program in three separate groups. Although there were no inclusion or exclusion criteria and screening for PTSD was not done using standardized methods, the children included in the study were directly exposed to assault and atrocities of war. The symptoms of PTSD were assessed using the PTSD Reaction Index and were lower after the program. A paired *t*-test was used to analyze pre and posttest differences in mean PTSD scores. There was a reduction in PTSD scores after the program (*P* < .001). Effect sizes were calculated using Cohen's *d* with values of 0.6, 2.1, and 2.4 for changes in pre and posttest measurements of Groups I, II, and III, respectively, indicating a moderate clinical difference (*d* = 0.5) in Group I and large clinical differences (*d* = 0.8) in groups II and III. The main limitation of this study was the absence of a control group. A later study by the same author randomized 82 adolescents who met the criteria for PTSD according to the Harvard Trauma Questionnaire [20]. They were randomly assigned to a 12 session mind body group program or a wait list control group. Here, again the mind body program included meditation, guided imagery, breathing techniques, self-expression through words, drawings and movement, autogenic training, biofeedback, and genograms. PTSD symptoms were assessed using the Harvard Trauma Questionnaire. The intervention group had significantly lower PTSD symptoms compared to the wait list control group. After the wait list control group received the intervention, their PTSD symptoms were reduced. This study meets most of the criteria for rigor as the sample size was adequate, participants were randomized to two interventions and the assessment tool was standardized for assessment of trauma.

A single group study was conducted on 122 preteen Israeli school children (ages between 8 and 12 years) affected by the second Lebanon war [21]. Those schools approved for participation by the school principals were included in this study. The yoga program consisted of 13 yoga sessions which included yoga postures and breathing practices conducted for 4 months. The assessments were made using the WHO Well-Being Index and a satisfaction questionnaire for the children. The teachers were given the Connor Abbreviated Questionnaire to assess the behavior of the students. The significant finding was improvement in attention span, restlessness and inattention based on Connor Abbreviated Symptoms rated by the teacher. There was no significant difference in the WHO Well-Being measures though the children expressed satisfaction with the yoga training they had received. The first and third scales are validated whereas the second (satisfaction questionnaire) was developed by the authors. The data were analyzed with non parametric statistics (i.e., Wilcoxon paired signed ranked test and Kruskall-Wallis test). Effect sizes were not calculated in this study. The advantage of the study is that the responses of the children as well as the observations of the teachers were taken into account using valid questionnaires. The disadvantages of the study were that there was no control group.

Children between 6 and 12 years of age (*n* = 226) who experienced the terrorist bomb explosion in Bali in 2002 and who were subsequently diagnosed with PTSD were studied [22]. The design was a longitudinal, quasi-experimental, single blind, and randomized control design. Forty-eight children received group Spiritual-Hypnosis Assisted Therapy (SHAT) while 178 did not. Posttraumatic stress disorder symptom scores reduced at the two year follow up after SHAT with 77.1 percent improvement compared to 24 percent in the control group.

In north eastern Sri Lanka, 71 children who were affected by the civil war and the tsunami participated in a study [23]. They were diagnosed for PTSD through an interview. The age range was between 8 and 14 years. Those with mental retardation, psychosis, or any psychological disorder were excluded from the study. They were randomized as two groups; one received six-session narrative exposure therapy for children (KIDNET) while the other group had six sessions of meditation-relaxation (MED-RELAX). Assessments were made after one and six months using the UCLA PTSD index for DSM IV in interview form. In addition, a 5-item scale was used to assess problems in functioning in different areas of children's lives (e.g., social relationships, family life, and general life satisfaction). Also children were asked 5 questions related to the tsunami (e.g., “Did you see the big wave close by?”). The data were analyzed with repeated measures ANOVA and chi-square tests to compare postvalues of the two groups for one and six months. Symptoms of PTSD were significantly reduced for both groups at one month and remained stable at six months. Within-group effect sizes based on Cohen's *d* were calculated, effect sizes for the KIDNET were 1.76 (CI 0.9–2.5) at one-month posttest and 1.96 (CI 1.1–2.8) at 6-month follow up while for MED-RELAX they were 1.83 (CI 0.9–2.6) and 2.20 (CI 1.2–3.0) at one and six months posttest, respectively. This study used standardized questionnaires and randomized participants to two interventions. The only possible limitation is the absence of a no intervention group. The study is particularly useful as the children had been traumatized by both war and tsunami and they improved with the interventions.

### 3.3. Interpersonal Violence

Usefulness of mindfulness meditation was evaluated for 97, fourth grade (mean age of 9.7 years, *n* = 55) and fifth-grade (mean age of 10.6 years, *n* = 42) students from Baltimore city [24]. Most of these children lived in low-income neighborhoods with high levels of violence. They were randomized as two groups, one received mindfulness meditation and yoga (*n* = 51) and the other was a control group (*n* = 46). Assessments were taken at the beginning and end of 12 weeks. Both groups were assessed using (i) the response to stress questionnaire (RSQ) from which the involuntary engagement coping scale was selected, (ii) the short mood and feelings questionnaire-Child version for depressive symptoms (SMFQ), (iii) the emotion profile inventory (EPI), and (iv) relations with peers and the school were assessed with the “People in my Life (PIML)” self-report measure. Data were analyzed using ANOVA for continuous variables and chi-square tests for categorical variables. The findings suggest that a mindfulness based intervention shows promise in reducing physical and cognitive ways of responding to stress among youth faced with violence in their daily life. Effect sizes were calculated for emotional profile positive affect (*d* = .04), Emotional profile negative affect (*d* = .13), SMFQ depression scores (*d* = .13), PIML trust in friends (*d* = .40), PIML communications with friends (*d* = .17), PIML Teacher Affiliation (*d* = .09), PIML dissatisfaction with teachers (*d* = .08), and RSQ (*d* = .83). The limitations include the fact that the recruitment may have resulted in highly motivated students and/or those with enthusiastic parents taking part. Also the self-report measures may have been influenced by various sources of bias.

### 3.4. Youth Incarcerated in a Correctional Facility

Youthful offenders are committed to legal custody and interventions are needed to help them cope with the stress and adjustment to the environment [25]. A randomized controlled trial was carried out on 28 girls between 12 and 16 years of age who were living in a community home as they had a history of committing legal offences [26]. The 28 girls were matched for age ±6 months and duration of stay in the community home ±2 months. Participants were then assigned to two groups randomly, namely, yoga or games. The yoga training included postures and guided relaxation sessions for 60 minutes daily for 5 days a week. At the end of 6 months, both groups' heart rate, breath rate and skin resistance were assessed to evaluate their physiological stress levels. Data were analyzed using Wilcoxon paired-sample test for pre and postcomparisons. Following 6 months of practice, both groups significantly reduced their heart rate, but the yoga group alone showed a decrease in breath rate. Effect sizes were not calculated in this study. The findings of the study were limited by (i) the small sample size, (ii) absence of tests for self reported stress and wellbeing, and (iii) the objective variables used were very simple and apart from the skin resistance do not directly relate to physiological stress [27]. 

The studies cited above have been summarized in Table 1.

## 4. Mechanisms Underlying the Improvement in the Psychological State Following Trauma with Yoga

Given the possibility of using yoga to positively modify the mental state following trauma, it is interesting to speculate about the mechanisms underlying the benefits seen. The amygdala is one of the main sites where alterations in the regulation of the serotonin transporter (5-HTT) may alter the stress response [28]. Based on a positron emission tomography scan, abnormally reduced amygdala 5-HT binding was found in PTSD and was associated with higher symptoms of anxiety and depression in posttrauma patients, particularly those with diagnosed PTSD. Hence, abnormal 5-HT signaling within neural systems possibly underlies threat detection and fear learning.

Whole blood serotonin levels and mood state changes were assessed before and after focused attention on Tanden breathing, which is a part of Zen meditation, in 15 healthy right-handed participants [29]. Recordings were made of the (i) electromyography (EMG) to monitor the abdominal muscle contraction, (ii) the electroencephalograph (EEG) from three scalp locations for monopolar recordings, (iii) electrooculography (EOG) and electrocardiography (ECG), (iv) near infrared spectroscopy (NIRS) using a 24-channel system, and (v) whole blood serotonin levels measured within 2 weeks of the experiment using standard high-performance liquid chromatography- (HPLC-) based methods. Different statistical tests were used for the different measures such as two way repeated measures ANOVA for EEG, one-way repeated measures ANOVA for serotonin levels, paired *t*-tests for the mood scores and one way repeated measures ANOVA for oxy-hemoglobin levels derived from the NIRS data. Effect sizes were not calculated in this study. During focused attention on Tanden breathing, there was a significant increase in the oxy-hemoglobin level in the anterior prefrontal cortex, increased alpha activity with decreased theta activity, and increase in whole blood serotonin levels correlated with increased alpha activity and reduced negative feelings.

Apart from changes in serotonin levels, animal models of depression often use traumatic experiences of pain, isolation, or social defeat to cause changes in mesolimbic and mesocortical dopamine systems, which alter cortical control of midbrain defenses [30]. Imbalance within the ascending dopaminergic tracts may cause rapid fluctuations in the level of arousal and in the associated mood, drive, and motivation. 

Stress reduction, positive affect, and levels of plasma catecholamines were assessed in 67 regular (range 3–144 months) meditators (aged 18–36 years) and 57 non-meditators (aged 19–37 years). The meditation practice was called “Brain Wave Vibration Mind Body Training” which is considered to change negative thoughts into positive ones [31]. The technique used natural rhythmic movements and hence it is a moving meditation. All participants were assessed using the stress response inventory, Positive Affect Negative Affect Scale (PANAS), as well as blood samples to estimate norepinephrine, epinephrine and dopamine levels using high-performance liquid chromatography (HPLC). Comparisons between the two groups were made with *t*-tests and Pearson's correlation coefficient was used to analyze the relationship between the two variables. Effect sizes were not calculated in this study. The meditation group had higher scores on positive affect and lower scores on negative affect compared with non meditators. Their plasma dopamine levels were also higher. The control group showed a negative correlation between stress and positive effect. A positive correlation was found between somatization of stress and norepinephrine/epinephrine and dopamine/epinephrine ratios. Hence in comparison with the control group, the regular meditators had lower stress, higher positive affect, and higher plasma dopamine levels.

While changes in serotonin and dopamine levels following meditation may explain at least in part the positive affect and reduction in stress following meditation the explanation for the anxiety lowering effect of the yoga practices may be related to another neurotransmitter, namely, Gamma Aminobutyric Acid (GABA). Two studies demonstrated that GABA-ergic activity increased after yoga practice. In the earlier study, 8 experienced yoga practitioners were compared with 11 non practitioners [32]. All subjects were evaluated using the Structural Clinical Interview for DSM-IV Axis I Disorders: Patient Edition (CID) and the Addiction Severity Index (ASI). Persons with contraindications for magnetic resonance evaluation were excluded from the study. The yoga group completed a 60-minute session which included yoga postures while the comparison subjects read periodicals and popular fiction during a 60-minute session. GABA-to-creatine ratio was measured using magnetic resonance spectroscopy imaging (MRSI) immediately before and after the intervention. The GABA levels increased by 27 percent in the yoga practitioners after the yoga session while the comparison group showed no change. In this study, effect size was not calculated. The second study by the same authors addressed the question of whether changes in GABA levels are specific to yoga or related to physical activity [33]. Participants were randomized to a yoga group (*n* = 19) or a metabolically matched physical exercise group (*n* = 15) for 60 minutes, 3 times a week for 12 weeks. The age range of the participants was 18–45 years. Magnetic resonance spectroscopy scans demonstrated that thalamic GABA levels increased in the yoga group and were positively correlated with improved mood. The acute changes in GABA levels in the yoga group approached significance (*P* = 0.09, *t*-test). In this study, also effect sizes were not calculated. The thalamic GABA level was associated with improved mood and decreased anxiety. These changes are usually obtained by pharmacological agents designed to improve mood and alleviate anxiety. Hence, this study was the first report of a behavioral intervention producing the same effect. 

These studies suggest that certain changes in neurotransmitters following yoga practice may be responsible for the improved psychological state in trauma victims who practiced yoga. However, neurotransmitters have not been measured in any of the studies on trauma victims who improved with yoga. Hence, this is a speculation.

For this review, we examined in detail eleven studies (indexed in PubMed) on people exposed to trauma who received yoga including meditation as an intervention. There was also a single group cross-sectional study conducted prior to one of the intervention studies. Hence, 12 studies were reviewed. Among them, there were 7 randomized controlled trials (RCTs), 4 single group studies, and the one cross-sectional single group survey referred to above. Even where RCTs were conducted studies were limited by factors such as small sample sizes and in a few cases use of assessment tools whose reliability and validity were not established. Hence, though yoga and other mind body interventions appear to be useful in reducing mental health disorders following trauma there is as yet no systematic randomized control trial which meets all the requirements to state that these interventions conclusively are useful in trauma management.

The last part of the article attempts to consider possible mechanisms underlying the improvement with yoga. However, since none of these studies were conducted in trauma survivors, they remain speculative, and a possible direction for future study.

## Figures and Tables

**Table 1:** Summary of the twelve studies reviewed

Sl. Number	Category of trauma	Sample: (1) Age, (2) Gender, (3) *n*	Study design	Assessment tools and their reliability/validity	Statistics	Effect sizes	Limitations
(1)	Natural disaster (tsunami) (Location: the Andaman islands) [14]	(1) 28–50 years (2) both genders (3) *n* = 47	Single group longitudinal design with before, after (7 day yoga program)	(i) Linear analog scales for fear, anxiety, disturbed sleep, and sadness (the reliability and validity has not been established), (ii) Heart rate variability from the electrocardiogram), (iii) breath rate and, (iv) skin resistance (ii), (iii), (iv) were recorded with a polygraph and were standardized	Paired *t*-tests	Not reported	(i) Absence of a control group, (ii) use of scales whose reliability and validity had not been established, and (iii) short duration of the intervention.

(2)	Natural disaster (tsunami) (Location: South-east coast of India) [15]	(1) 18–65 years (2) Both genders (3) *n* = 183	Allocation (non random) to 3 groups (a) yoga breath intervention, (b) yoga breath intervention followed by trauma reduction exposure technique, and (c) a wait list control. (d) Longitudinal assessments before, after (24 weeks) as well as intermittently	(i) The PTSD check list-17 (PCL-17). (ii) Beck depression inventory (BDI-21) reliability and validity are established	Three factor ANOVA, with *post hoc* analysis using Newman Keuls tests	Not reported	Allocation to the 3 groups was not random

(3)	Natural disaster (Hurricane Katrina) (Location: New Orleans, U.S.A.) [16]	(1) 31–67 years (2) Both genders (3) *n* = 20	(i) Single group (ii) longitudinal assessments before, after (8 weeks of intervention)	(i) PTSD checklist-Specific version (PCL-S) (ii) Centre for (CES-D) (iii) State subscale of STAI (STAI-S) (iv) a follow up questionnaire (i), (ii), and (iii) are reliable and valid	Intention-to-treat regression analysis and one sample *t*-tests	PCL-S (*d* = .38) CES-D (*d* = .12) STATE-S (*d* = .45)	(i) Small sample size and (ii) absence of a control group

(4)	Natural disaster (floods) (Location: Bihar, India) [17]	(1) 15–85 years (2) Both genders (3) *n* = 1289	Cross sectional single group study evaluating risk for PTSD and depression in different age groups	Screening questionnaire for disaster mental health (SQD) with known reliability and validity	Two factor ANOVAs followed by *post-hoc* analyses	Not reported	Confounding variables which could influence susceptibility other than age and gender were not reported

(5)	Natural disaster (floods) (Location: Bihar, India) [18]	(1) 20–40 years (2) Males (3) *n* = 22	(i) Randomized controlled study (ii) Longitudinal with before, after assessment after 7 days of intervention	(i) Linear analog scales to assess fear, anxiety, disturbed sleep and sadness, (ii) heart rate variability based on electrocardiogram, and (iii) breath rate. reliability and validity of linear analog scales are not established and (ii) and (iii) were recorded on a polygraph with standardized methods	Paired *t*-tests	Not reported	(i) Small sample size and (ii) use of analog scales which are not valid

(6)	Exposure to combat and terrorism (Location: Kosovo) [19]	(1) 12 to 19 years (2) Both genders (3) *n* = 139	(i) Single group. (ii) Longitudinal with before, after (6 weeks) assessments	(i) PTSD reaction Index With known reliability and validity	Paired *t*-tests RMANOVA for one group and the subset of another group	Effect size for group 1 (*d* = 0.5) and group II and II (*d* = 0.8)	Absence of a control group

(7)	Exposure to combat and terrorism (Location: Kosovo) [20]	(1) 12–19 years (2) Both genders (3) *n* = 82	(i) Randomized control study (ii) Longitudinal before, after (3 months)	Harvard trauma questionnaire (valid and reliable)	Repeated measures ANOVA	Not obtained possibly reported	NIL

(8)	Exposure to combat and terrorism (Location: Israel) [21]	(1) 8–12 years (2) Both genders (3) *n* = 122	(i) Single group (ii) Longitudinal before, after (4 months)	(i) WHO well being index (ii) A satisfaction questionnaire. (iii) Connor abbreviated questionnaire (rated by the teacher). (i) and (iii) are reliable and valid	(i) Wilcoxon paired signed ranked test (ii) Kruskall Wallis test	Not reported	Absence of a control group

(9)	Exposure to combat and terrorism (Location: Bali) [22]	(1) 6–12 years (2) Both genders (3) *n* = 226	(i) Single blind, randomized control design. (ii) Longitudinal before, after (2 year follow up)	Not obtained	Not obtained	Not obtained	Numbers in the 2 groups were unequal (i.e., 48 in the SHAT group compared to 178 in the control) this is not usual in a randomized control design

(10)	Exposure to combat and terrorism as well as tsunami (Location: Sri Lanka) [23]	(1) 8–14 years (2) Both genders (3) *n* = 71	Randomized to 2 interventions, before, after (1, 6 months)	(i) (UCLA PTSD index for DSM-IV. (ii) 5 item scale for satisfaction. (iii) 5 item scale related to the tsunami. (i) and (ii) had established reliability and validity	Repeated measures ANOVA and chi-square tests	Effect Sizes (i) KIDNET 1.76 (one month post test), 1.96 (6 months post test) (ii) MED-RELAX 1.83 (one month post test), 2.20 (6 month post test)	(i) Absence of a no intervention control group. (ii) The third questionnaire (related to the tsunami) was not standardized

(11)	Interpersonal violence (Location: Baltimore City, U.S.A.) [24]	(1) 9–11 years (2) Both genders (3) *n* = 97	Randomized control before, after (12 weeks) assessments	(i) Response to stress questionnaire. (ii) The short mood and feelings questionnaire: Child version for depressive symptoms. (iii) Emotional profile inventory (iv) “People in my life self report measure”	(i) ANOVA for continuous variables. (ii) chi-square tests for categorical variables	Effect sizes were calculated for Emotional profile positive affect (*d* = .04), Emotional profile negative affect (*d* = .13), SMFQ depression scores (*d* = .13), PIML Trust in Friends (*d* = .40), PIML Communications with Friends (*d* = .17), PIML Teacher Affiliation (*d* = .09), PIML Dissatisfaction with Teacher (*d* = .08) and RSQ (*d* = .83)	Recruitment may have involved highly motivated students with enthusiastic parents (the self-report measures may have been influenced by bias

(12)	Youth in a correctional facility (Location: Bangalore, India) [26]	(1) 12–16 years (2) Females (3) *n* = 28	Matched pair allocation to intervention and control groups (pairs were matched for age and duration of stay in the community home), before, after (6 months) assessments	(i) Heart rate from the electrocardiogram. (ii) Breath rate from a respirogram. (iii) Skin resistance. The 3 variables were recorded with a polygraph using standard methods	Wilcoxon paired—sample tests	Not reported	(i) The small sample size. (ii) absence of tests for self reported stress, and (ii) simplistic variables used are inadequate to assess physiological stress
